# Structural and Functional Basis of JAMM Deubiquitinating Enzymes in Disease

**DOI:** 10.3390/biom12070910

**Published:** 2022-06-29

**Authors:** Xin Pan, Sihua Wu, Wenping Wei, Zixuan Chen, Yong Wu, Kaizheng Gong

**Affiliations:** 1 Department of Cardiology, The Affiliated Hospital of Yangzhou University, Yangzhou University, Yangzhou 225000, China; xinpanphd@163.com (X.P.); weiwp_yzu@163.com (W.W.); is_zixuanchen@163.com (Z.C.); 13852786827@163.com (Y.W.); 2 State Key Laboratory of Biochemical Engineering, Institute of Process Engineering, Chinese Academy of Sciences, Beijing 100864, China; defupai@163.com; 3 Division of Molecular Science, Graduate School of Science and Technology, Gunma University, Kiryu 376-8515, Gunma, Japan

**Keywords:** deubiquitinating enzymes, JAMMs, structural basis, catalytic mechanism, functions, inhibitors

## Abstract

Deubiquitinating enzymes (DUBs) are a group of proteases that are important for maintaining cell homeostasis by regulating the balance between ubiquitination and deubiquitination. As the only known metalloproteinase family of DUBs, JAB1/MPN/Mov34 metalloenzymes (JAMMs) are specifically associated with tumorigenesis and immunological and inflammatory diseases at multiple levels. The far smaller numbers and distinct catalytic mechanism of JAMMs render them attractive drug targets. Currently, several JAMM inhibitors have been successfully developed and have shown promising therapeutic efficacy. To gain greater insight into JAMMs, in this review, we focus on several key proteins in this family, including AMSH, AMSH-LP, BRCC36, Rpn11, and CSN5, and emphatically discuss their structural basis, diverse functions, catalytic mechanism, and current reported inhibitors targeting JAMMs. These advances set the stage for the exploitation of JAMMs as a target for the treatment of various diseases.

## 1. Introduction

**Figure 1:**
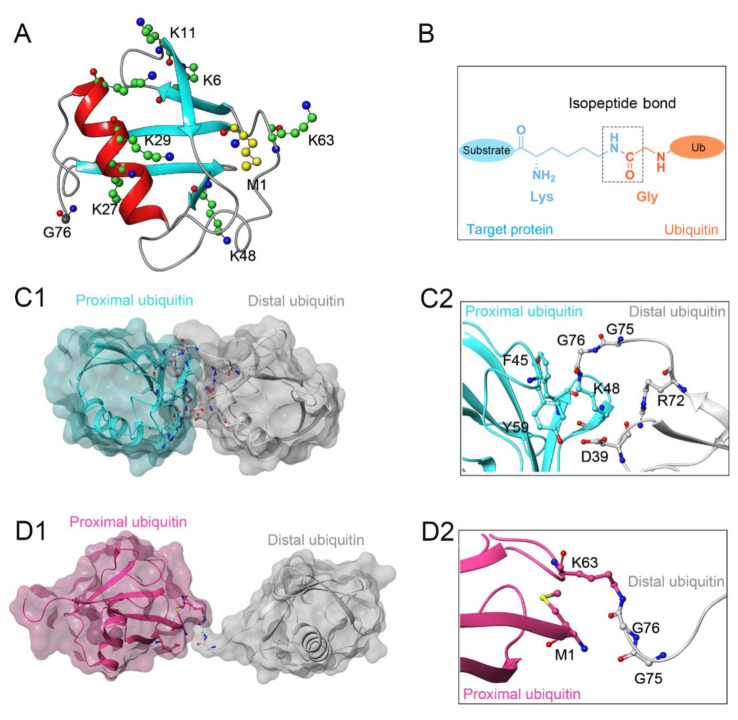
(**A**) Crystal structure of human ubiquitin (PDB ID: 1UBQ). Seven lysine residues and an N-terminal methionine residue are colored green and yellow, respectively. (**B**) The isopeptide bond between the ubiquitin glycine residue (orange) and the target protein lysine residue (blue). (**C1**,**C2**) The overall structure and local conformation of K48-linked polyubiquitin chains (PDB ID: 1TBE). (**D1**,**D2**) The overall structure and local conformation of K63-linked polyubiquitin chains (PDB ID: 3HM3). The distal and proximal ubiquitin are colored gray and cyan, respectively.

Protein ubiquitination, defined as a process that covalently conjugates ubiquitin to the target protein, is one of the most powerful post-translational modifications regulating virtually all cellular processes, such as cell death, cell cycle, and DNA repair [1,2,3,4]. Ubiquitin is a 76-amino-acid, 8-kDa polypeptide with a conserved sequence that is present universally and ubiquitously in eukaryotes [5,6]. Full-length ubiquitin contains eight ubiquitination sites, including seven lysine residues (K6, K11, K27, K29, K33, K48, K63) and an N-terminal methionine residue (M1) (Figure 1A) [7,8]. Under the sequential action of ubiquitin-activating enzymes (E1), ubiquitin-conjugating enzymes (E2), and ubiquitin ligases (E3), an isopeptide linkage is formed between the carboxyl group of the ubiquitin C-terminal glycine and the ε-amino group of the target protein lysine (Figure 1B) [9,10]. Then, the Gly 76 of additional ubiquitin molecules (called distal ubiquitin) can be covalently attached to the ubiquitination sites in ubiquitin itself (called proximal ubiquitin), to produce polyubiquitin chains [11]. As a result, various types of protein ubiquitination are formed, which determine the fate of ubiquitinated substrates [6,12]. For example, polyubiquitin chains linked via the K48 of internal ubiquitin groups are used for protein degradation signaling by the ubiquitin-proteasome system (Figure 1C1,C2), whereas K63-linked polyubiquitin chains, presenting different architecture, play proteasome-independent roles in various intracellular events, such as inflammatory signaling, DNA repair, ribosomal protein synthesis, endocytosis, and vesicular trafficking (Figure 1D1,D2) [13,14,15]. Additionally, some other ubiquitin-like modifications, such as small ubiquitin-like modifier (SUMO), neuronal precursor cell-expressed developmentally downregulated protein 8 (NEDD8), and interferon stimulated gene 15 (ISG15), can also be ligated to target proteins in a process similar to ubiquitylation, mostly to provide nondegradative signals [16].

To date, the human genome encodes nearly 600 E3 ligases while only approximately 100 deubiquitinating enzymes (DUBs) have been identified, clustered in the following 7 families: 56 ubiquitin-specific peptidases (USPs), 17 ovarian tumor proteases (OTUs), 12 JAB1/MPN/Mov34 metalloenzymes (JAMMs), 5 motif interacting with ubiquitin-containing novel DUB family proteases (MINDYs), 4 ubiquitin C-terminal hydroxylases (UCHs), 4 Machado-Josephin domain proteases (MJDs), and 1 zinc finger-containing ubiquitin peptidase 1 (ZUP1) [17,18,19]. Six of these seven families are cysteine proteases, whereas only the JAMM family are zinc-dependent metalloproteinases. In the case of cysteine protease DUBs, the catalytic domains contain a highly conserved catalytic triad comprising cysteine and nearby histidine and aspartate residues [8,20]. In contrast, metalloprotease DUBs coordinate Zn^2+^ ion with histidine, aspartate, and serine residues to attack the isopeptide bond by activating a water molecule [21].

Dysfunction of the ubiquitin system, especially DUBs, has been recognized as a contributing factor in the origin of many human diseases, such as cancer, inflammatory diseases, and neurological diseases [22,23]. Notably, there has been a recent expansion of drug discovery programs targeting JAMMs. Unlike the large number of cysteine protease DUBs (~90), as few as 12 JAMMs have been identified in the human genome, among which only 7 (AMSH, AMSH-LP, BRCC36, eIF3h, Rpn11, MYSM1, and CSN5) exhibit isopeptidase activity toward ubiquitin chains [17,24]. Furthermore, multiple JAMM-related signaling pathways, such as DNA damage control (BRCC36) [25], endocytosis (AMSH, AMSH-LP) [26,27], protein biosynthesis (eIF3h) [28], and protein degradation (Rpn11, CSN5) [29], have been confirmed to be associated with numerous diseases, including tumorigenesis and immunological and inflammatory disorders. The much fewer numbers, distinct catalytic mechanism, and, specifically, association with diseases render JAMMs a new class of potential drug targets [30]. To gain an in-depth understanding of JAMMs, this review emphatically discusses the structural basis, catalytic mechanism, and diverse functions with a focus on JAMM family proteins, including AMSH, AMSH-LP, BRCC36, Rpn11, and CSN5. We also summarize the current reported inhibitors targeting JAMM family members.

## 2. Structural Characteristic of JAMMs

**Figure 2:**
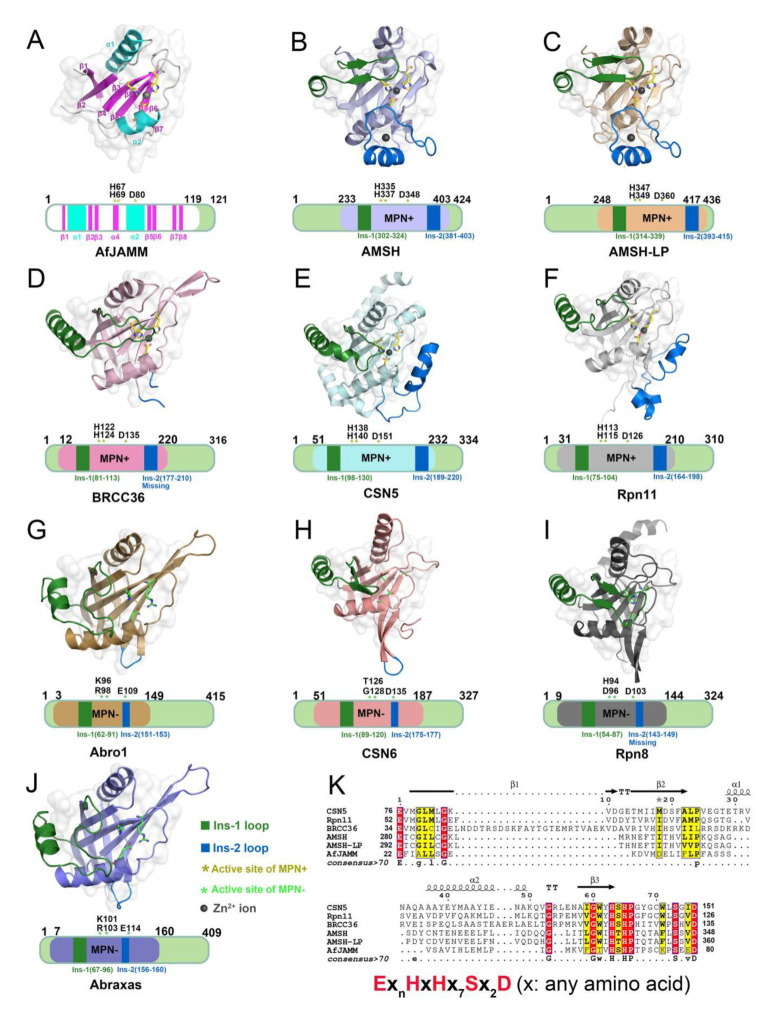
Structural characteristics of JAMM MPN domain mentioned in this review. (**A**) Crystal structure of AfJAMM (PDB ID: 1R5X). AfJAMM has a typical MPN domain containing an eight-stranded β sheet (β1–β8) (fuchsia), a long α helix (α1), and a short α helix (α2) (cyan). (**B**–**J**) Crystal structure of AMSH (PDB ID: 3RZU) (light blue), AMSH-LP (PDB ID: 2ZNV) (wheat), BRCC36 (PDB ID: 6H3C) (light pink), CSN5 (PDB ID: 4F7O) (pale cyan), Rpn11 (PDB ID: 4O8X) (gray), Abro1 (PDB ID: 6H3C) (sand), CSN6 (PDB ID: 4D10) (salmon), Rpn8 (PDB ID: 4O8X) (light black), and Abraxas (PDB ID: 6GVW) (slate) MPN domain. All the Ins-1 and Ins-2 loop are colored deep green and blue, respectively. The yellow and green asterisks represent active sites of MPN+ and MPN–, respectively. The black round represents Zn^2+^ ion. (**K**) The zinc-coordinating JAMM motif of MPN+ (Ex_n_HxHx_7_Sx_2_D) (where x is any amino acid residue).

The MPN (Mpr1/Pad1 N-terminal) domain is a striking characteristic of the JAMM family. In 2004, Ambroggio et al. first reported the crystal structure of the MPN domain protein (PDB ID: 1R5X) from a prokaryotic organism *Archaeoglobus fulgidus* AF2198 (AfJAMM) [31]. They revealed that the MPN domain of AfJAMM consisted of an eight-stranded β sheet (β1–β8), flanked by a long α helix (α1) between the first and second strand, and a short α helix (α2) between the fourth and fifth strand (Figure 2A). Subsequently, an increasing number of crystal structures were resolved and the MPN domain proteins could then be further divided into two subfamilies: (1) the MPN+ family, with isopeptidase activity, characterized by a zinc-coordinating JAMM motif (Ex_n_HxHx_7_Sx_2_D) (where x represents any amino acid residue) (Figure 2K); and (2) the MPN– family, without catalytic activity, serving as scaffolds in some JAMM multi-subunit complexes [32,33,34].

Most JAMMs possess two unique insertions, referred to as Ins-1 and Ins-2, which are considered to play important roles in the recognition and binding of ubiquitinated protein substrates [24]. The Ins-1 segment forms one ridge of the substrate-binding groove to assist in the proper positioning of the C-terminal ubiquitin tail for catalysis while the Ins-2 region contributes to the productive substrate positioning [35].

**Figure 3:**
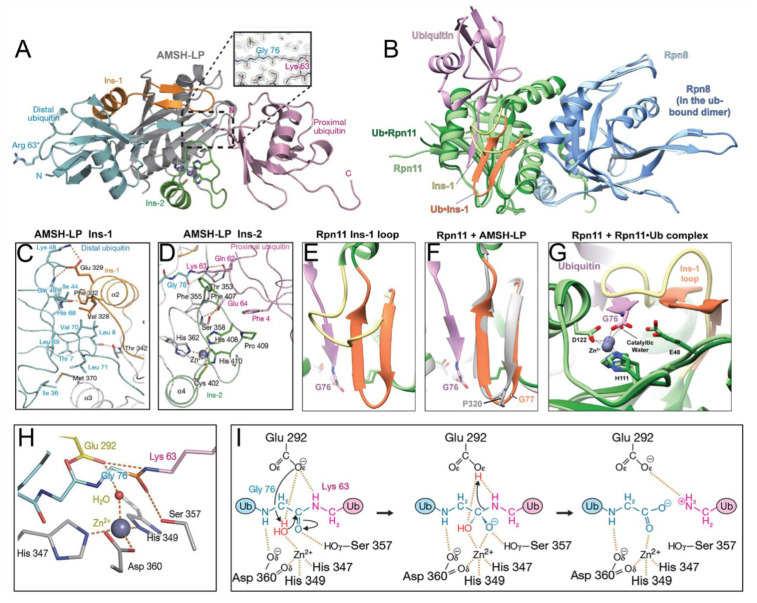
(**A**) Crystal structure of the AMSH-LP DUB domain in complex with K63-linked polyubiquitin chains (PDB ID: 2ZNV). The JAMM core, Ins-1, and Ins-2 are colored gray, orange, and green, respectively. The proximal and distal ubiquitins are colored pink and cyan, respectively. (**B**) Superimposition of the crystal structures for the Rpn11-Rpn8 heterodimer with ubiquitin (PDB ID: 5U4P, green, blue, and pink, respectively) and the ubiquitin-free Rpn11-Rpn8 heterodimer (PDB ID: 4O8X, light green and light blue). (**C**,**D**) The interfaces between AMSH-LP and the distal/proximal ubiquitin. (**E**) Close-up view of the Ins-1 loop in the ubiquitin-free state (tan) and the ubiquitin-bound state (orange). (**F**) Superimposition of the Ins-1 loop of Rpn11 in the ubiquitin-bound state with the Ins-1 loop of AMSH-LP in the ubiquitin-free state (PDB ID: 2ZNR). The Ins-1 loop of AMSH-LP always occupies the β-hairpin conformation, even in the absence of ubiquitin. (**G**) Close-up view of the superimposed active sites of Rpn11 in the ubiquitin-free (light green) and ubiquitin-bound (green) states. (**H**) The catalytic domains of AMSH-LP. The catalytic Zn^2+^ ion is complexed to His 347, His 349, and Asp 360 and an activated water molecule to attack the isopeptide bond of ubiquitin chains. (**I**) Take AMSH-LP as an example, the schema represents a proposed catalytic mechanism of JAMMs. Panels **A**, **C**, **D**, **H**, **I**, and **B**, **E**–**G** are adapted with permission from [26,39], respectively. Copyright 2008 Springer Nature, and copyright 2017 Elsevier.

In the previously reported JAMM crystal structures, the Ins-1 segment has some degree of conservation; in AMSH-LP, AMSH, CSN6, and Rpn8, the Ins-1 segment contains a long helical portion and a β-hairpin while in BRCC36, Abro1, Abraxas, CSN5, and Rpn11, the segment adopts a helical portion and long loop (Figure 2B–J). Using the AMSH-LP^E292A^-ubiquitin complex (PDB ID: 2ZNV) from *Homo sapiens* as an example, the C-terminal tail of the distal ubiquitin moiety binds the active site cleft and is stabilized with the Ins-1 segment by extensive hydrophobic interactions and hydrogen bonds, thus facilitating a scissile isopeptide bond for hydrolysis (Figure 3A,C) [26]. Furthermore, evidence has suggested that the Ins-1 region is flexible in the binding of distal ubiquitin, as it undergoes conformational transitions between inactive and active states [24,36,37]. These states have been confirmed by the crystal structure of the dimeric Rpn11-Rpn8 complex (PDB ID: 4O8X) and the ternary Rpn11-Rpn8-ubiquitin complex (PDB ID: 5U4P) from *Saccharomyces cerevisiae*; the Ins-1 loop of Rpn11 is in the inactive state in the former and in the active state in the later (Figure 3B) [38,39,40]. In the closed state of Ins-1, this loop blocks access of the C-terminus of ubiquitin to the active site. In the open state, Ins-1 is not simply displaced but undergoes a conformational transition to a β-hairpin, thus favoring the proper positioning and cleavage of the C-terminal ubiquitin tail (Figure 3E–G). Similar conformational changes are observed in the crystal structures of the CSN5 monomer (PDB ID: 4F7O) and CSN5 with inhibitor CSN5i-3 complex (PDB ID: 5JOG) from *H. sapiens* [41,42]. The Ins-1 loop in the monomer is found to be in a closed conformation, blocking the exposure of the binding site, whereas the complex takes on an open conformation as the Ins-1 loop is pointing away from the catalytic site. Therefore, it is speculated that once a ubiquitinated substrate or ligand enter the catalytic site, the conformation state of the Ins-1 loop will be changed to help position the substrate [24,43].

Unlike the relatively conserved Ins-1 segment, the Ins-2 exhibits large differences among the JAMMs. Except for the unresolved Ins-2 structure of BRCC36, almost all of the MPN+ members have a long loop with helix while the Ins-2 structure of MPN− members is an extremely short loop. Specifically, the Ins-2 segment of AMSH-LP consists of a loop and helical portion, which forms a flap structure near the active site and is stabilized by the coordination of a non-catalytic Zn^2+^ ion. The interaction of Ins-2 with the Gln 62 and Glu 64 of the proximal ubiquitin is considered to facilitate the correct orientation, thus dictating substrate specificity for K63-linked ubiquitin chains (Figure 3D) [26]. Compared to AMSH-LP, the Ins-2 segment of Rpn11 has an entirely different function, rendering it highly promiscuous [38,40]. In the crystal structure of the 26S proteasome complex, the Ins-2 loop of Rpn11 is disordered and interacts with another subunit Rpn2, preventing contact with the proximal ubiquitin. For this reason, Rpn11 is capable of cleaving multiple types of ubiquitin chains, which is essential for the efficient and non-discriminative ubiquitin removal from hundreds of different substrates during proteasomal degradation [38,40]. Accordingly, it is likely that the Ins-2 region plays a leading role in determining ubiquitin-ubiquitin linkage-type specificity.

## 3. Catalytic Mechanism of JAMMs

So far, 7 of the 12 JAMMs (AMSH, AMSH-LP, BRCC36, eIF3h, Rpn11, CSN5, and MYSM1) in the human genome belong to the MPN+ subfamily and have DUB activity toward proteins while the remaining 5 JAMMs (Abraxas, Abro1, CSN6, eIF3f, and Rpn8) belong to the MPN− subfamily [44,45]. Interestingly, most of these JAMMs require the formation of multi-subunit complexes to exert their isopeptidase activities, including Rpn11 and Rpn8 of the 26S proteasome [29], CSN5 and CSN6 of the COP9 signalosome [46], eIF3f and eIF3h of the human translation initiation factor 3 (eIF3) [47], BRCC36 and Abraxas of the BRCA1-A complex [48], and BRCC36 and Abro1 of the BRISC complex [49]. There are, of course, exceptions, such as AMSH and AMSH-LP, which can cleave K63-linked ubiquitin chains independent of protein partners [50]. Sato et al. resolved the crystal structure of AMSH-LP^E292A^-ubiquitin complex (PDB ID: 2ZNV) from *H. sapiens* and proposed the catalytic mechanism of JAMMs, which was probably similar to that of thermolysin (Figure 3H) [26,51].

First, the zinc-bound catalytic water is deprotonated by an active site Glu 292 and subsequently performs a nucleophilic attack on the substrate peptide carbonyl. Then, the negative charge on the peptide carbonyl oxygen is stabilized by the Zn^2+^ ion and His 347, His 349, Ser 357, and Asp 360 while the positive charge on the amide nitrogen is stabilized by Glu 292. The reaction then proceeds through an oxyanion tetrahedral intermediate and a second transition state, which results in the cleavage of the peptide N-C bond. With the proton transferring from the amide nitrogen to water, the cleavage of the peptide bond is ultimately completed (Figure 3I) [26]. Although the whole amino acid sequences of these seven MPN+ members are highly divergent, the catalytic core region is completely conserved, suggesting that they might employ identical catalytic mechanisms [30].

## 4. Structural and Functional Basis of JAMMs

### 4.1. Functional Basis of AMSH in Receptor Endocytosis

It has recently been shown that AMSH plays a significant role in regulating the endosomal sorting of many cell-surface receptors, which is a highly regulated process for maintaining cellular homeostasis and generating adaptive responses to external stimuli [52,53]. Typically, the endocytic trafficking process involves the internalization, endosomal sorting, and lysosomal degradation of cell-surface receptors and is strictly executed by the endosomal sorting complexes required for transport (ESCRT), consisting of at least five macromolecular assemblies termed ESCRT-0, ESCRT-I, ESCRT-II, and ESCRT-III and vacuolar sorting protein 4 (Vps4) [54,55,56]. It is during this process that AMSH can interact with the components ESCRT-0 and ESCRT-III and so affect the fate of receptors [16].

Several studies have documented the crucial role of AMSH-mediated deubiquitination in the trafficking of endocytosed receptors, such as receptor-tyrosine kinase epidermal growth factor receptor (EGFR), G protein-coupled receptors (GPCRs), connexins 43 (connexin Cx43), and the inflammasome component NACHT, LRR, and PYD domain-containing protein (NALP7) (Table 1) [57,58,59,60,61,62]. For example, the E3-ligase c-Cbl has been shown to promote lysosomal degradation of the K63 ubiquitylated EGFR [63] while AMSH opposes this action and promotes EGFR recycling, thus regulating the balance of the intracellular EGFR content [59]. In another study, Ribeiro-Rodrigues et al. demonstrated that AMSH could protect gap junctions from degradation by mediating the deubiquitination of Cx43 to regulate intercellular communication [60]. By linking the DUBs to immune regulation, Mallampalli et al. found that AMSH cleaved K63-linked ubiquitin from NALP7 to increase its intracellular content, leading to inflammasome-dependent IL-1β cleavage and release [62]. For some important GPCRs, including chemokine receptor CXCR4, protease-activated receptor 2 (PAR_2_), and δ-opioid receptor (DOR), AMSH has been reported to regulate their stability and trafficking, as the loss of AMSH catalytic activity can significantly alter the steady-state level of GPCRs [57,58,61]. Overall, AMSH-mediated receptor endocytosis is accomplished through the recognition of specific ubiquitination patterns, specifically multi-monoubiquitination and K63-linked polyubiquitination.

### 4.2. Structural Basis of AMSH

**Figure 4:**
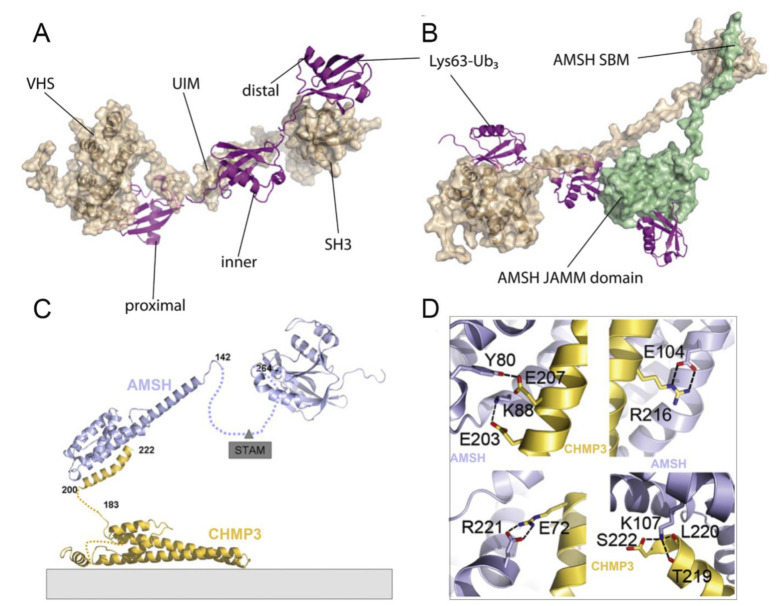
(**A**,**B**) A possible structural model for AMSH-STAM-ubiquitin complex. The AMSH, STAM, and ubiquitin are colored green, sand, and fuchsia, respectively. (**C**) The molecular model of AMSH in complex with CHMP3. (**D**) Close-up of the CHMP3–AMSH interactions group (PDB ID: 2XZE). The AMSH and CHMP3 are colored light blue and yellow, respectively. Hydrogen bonds and salt bridges along the CHMP3 helical segment mediate high-affinity interaction. Panels **A**, **B** and **C**, **D** are adapted with permission from [79,82], respectively. Copyright 2016 Elsevier, and copyright 2011 Elsevier.

ESCRT-0 mainly comprises two subunits, Hrs and STAM [76]. The interaction of ESCRT-0 and AMSH is achieved through the binding of the SH3 domain of STAM with the SH3-binding motif (SBM) of AMSH [77]. Based on the NMR structure of AMSH and STAM complex (PDB ID: 5IXF) from *H. sapiens*, Hologne et al. considered that the interaction of AMSH-SBM and STAM-SH3 contributed to the correct positioning of polyubiquitin chains toward AMSH before cleavage [78,79]. Subsequently, they proposed a structural model for AMSH-STAM-ubiquitin complex, in which the activation of AMSH was allowed by facile, simultaneous binding to two ubiquitin groups in a polyubiquitin substrate: one (distal ubiquitin) by the catalytic domain of the AMSH and the other (proximal ubiquitin) by the UIM domain of STAM (Figure 4A,B). Such a binding mode would stabilize the ubiquitin chain in a productive orientation, resulting in an enhancement of the DUB activity [78,79]. Meanwhile, another ubiquitin-binding domain of STAM, the VHS domain, is shown to enhance the cleavage of ubiquitin chains composed of more than two ubiquitin molecules. The absence of the VHS domain removes the specificity toward tri-ubiquitin, suggesting that this domain is essential for specificity toward longer chains [79].

The assembly of ESCRT-III is a highly ordered process involving seven charged multivesicular body protein (CHMP) subunits (CHMP 1-7) [80]. The N-terminus of AMSH contains nuclear localization signal and the microtubule-interacting and transport (MIT) domain, which has been shown to interact with several CHMPs, including CHMP1, CHMP2, and CHMP3 [53,81]. However, in the case of the crystal structure of an N-terminal fragment of AMSH (AMSHΔC) in complex with the C-terminal region of CHMP3 (CHMP3ΔN) (PDB ID: 2XZE) from *H. sapiens*, Solomons et al. found a higher affinity between CHMP3 and AMSH, indicating that AMSH might employ different interaction surfaces for these CHMPs [82]. They also found that CHMP3ΔN interacted with the MIT domain of AMSH involving multiple amino acids, including a hydrogen bond between Glu 207 and Tyr 80 and salt bridges between Glu 203-Lys 88, Arg 216-Glu 104, and Arg 221-Glu 72. The CHMP3 C-terminal residue Ser 222 was capped by AMSHΔC Lys 107, contributing to the formation of a salt bridge with the carboxyl group of Ser 222 and hydrogen bonds with the carbonyls of Thr 219 and Leu 220 (Figure 4D). Given this, an appropriate molecular model of the AMSH-CHMP3 complex is proposed, in which AMSH is first recruited to membranes early in the ESCRT pathway via ESCRT-0 STAM (Figure 4C) [83]. Then, the helical extension of the AMSH MIT domain can serve as a long arm to position the DUB activity > 20 nm away from the ESCRT-III polymer, thus reaching into the vesicle formed by ESCRT-I and ESCRT-II for receptor deubiquitination [26,82].

### 4.3. Comparison of AMSH and AMSH-LP

Interestingly, AMSH has a close homolog AMSH-LP (AMSH-like protein) [35]. Although the entire amino acid sequences of AMSH and AMSH-LP are only 54% identical, their three-dimensional structures exhibit extremely high similarity [16,26]. Especially, their catalytic domains and residues involved in proximal ubiquitin recognition are completely conserved [50,84]. However, AMSH-LP lacks several key features and presents some significant differences in the residues used for interaction with the distal ubiquitin [85]. Besides, AMSH contains an SBM domain, which interacts with the STAM of ESCRT-0 while a functional SBM is lost in human AMSH-LP [16]. By further exploring the differences in the properties, Davies et al. found that the catalytic domain of AMSH was thermodynamically less stable than that of AMSH-LP. They suggested that a more stable protein (AMSH-LP) was likely to have improved close packing of side chains, making it more rigid, whereas a less stable protein (AMSH) would be more plastic, which may make AMSH more suitable for interacting with other proteins, such as ESCRT-0 and ESCRT-III [85].

### 4.4. Functional Basis of BRISC in Inflammation, Immune Response, Mitosis, and Hematopoiesis

**Figure 5:**
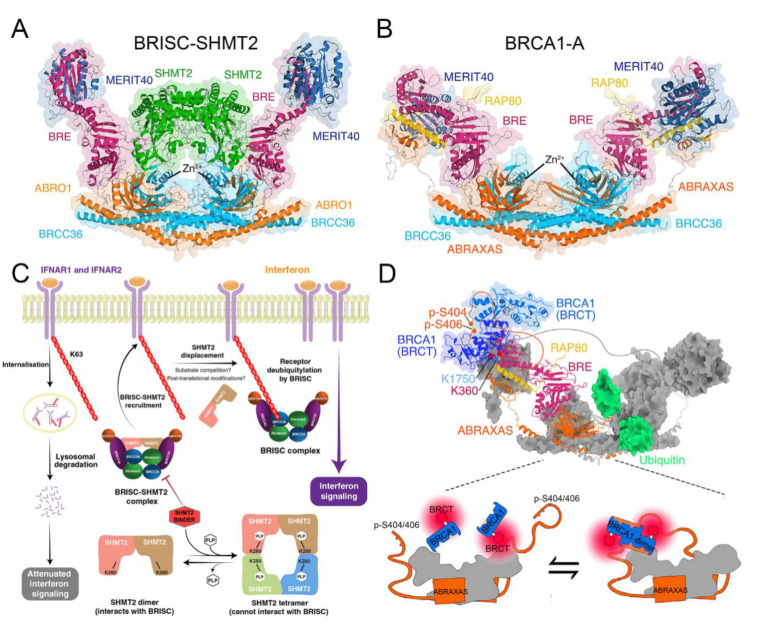
(**A**) Cryo-EM structure of the BRISC-SHMT2 complex (PDB ID: 6H3C). The BRCC45/BRE, MERIT40, BRCC36, Abro1/Abraxas2/KIAA0157, and SHMT2 are colored red, blue, cyan, orange, and green. (**B**) Cryo-EM structure of the BRCA1-A complex (PDB ID: 6GVW). The BRCC45/BRE, MERIT40, BRCC36, Abraxas, and RAP80 are colored red, blue, cyan, orange, and yellow. (**C**) Proposed model of BRISC-SHMT2 regulation of cytokine signaling using interferon as an example. The binding of PLP and SHMT2 regulates the BRISC–SHMT2 interaction and immune signaling in cell. Only the inactive SHMT2 dimer, but not the active PLP-bound tetramer, is able to bind and inhibit BRISC. (**D**) The model of the BRCA1-A-BRCA1 high-affinity complex. When DNA damage occurs, the S404 and S406 sites of Abraxas (orange) are doubly phosphorylated (p-S404/p-S406) and provide a high-affinity docking cradle for BRCA1 (blue), thereby inducing the formation of BRCA1 dimerization at the periphery of the BRCA1-A arc (gray). Panels **A**, **B**, **D**, and **C** are adapted with permission from [87,88], respectively. Copyright 2019 Elsevier, and copyright 2019 Springer Nature.

Unlike the monomer of AMSH or AMSH-LP with inherent DUB activity, BRCC36 must form a complex with other subunits to specifically cleave K63-linked polyubiquitin chains. In the cytoplasm, BRCC36 and the subunits BRCC45/BRE, MERIT40, and Abro1/Abraxas2/KIAA0157 form a BRISC (BRCC36 isopeptidase complex) complex (Figure 5A) [86]. By regulating the K63-linked ubiquitination of substrate proteins, the BRISC complex has been confirmed to play significant roles in various signaling pathways, including inflammation, immune response, mitosis, and hematopoiesis (Table 1) [64,65,66,67,68].

It is well known that the NLRP3 inflammasome is a multi-subunit complex that consists of NLRP3, ASC, and pro-caspase-1, which mediates the activation of caspase-1 and the secretion of mature IL-1β and IL-18 [89]. Recently, Py et al. found that extracellular ATP-induced IL-1β secretion and caspase-1 maturation could be significantly inhibited by reducing the expression of BRCC36 in macrophages, suggesting that BRCC36 may be a critical regulator of NLRP3 activity [64]. Based on these findings, Ren et al. further investigated the mechanism and revealed that lipopolysaccharide (LPS) priming induces Abro1 binding to NLRP3 in an S194 phosphorylation-dependent manner, subsequently recruiting BRISC to remove K63-linked ubiquitin chains from NLRP3, thereby activating NLRP3 and promoting inflammasome assembly [90,91]. The important antiviral factors type I interferons (IFNs) represent another typical BRISC-mediated example. The cellular response to IFNs is regulated by the abundance of the IFNAR1/2 receptor on the cell surface, which can be endocytosed and degraded after K63-linked ubiquitination [92]. However, the DUB activity of BRISC counteracts the degradation by deconjugating the ubiquitin chains of IFNAR1/2, promoting the cellular response to IFNs [65]. With respect to the key regulator of viral transcription HIV-1 Tat, it can be marked by K63-linked ubiquitin chains for selective autophagy and coupled lysosomal destruction, thus causing the provirus to persist for long periods [93]. Recent studies have shown that serine hydroxymethyltransferase 2 (SHMT2) and BRISC cooperate to rescue Tat from destruction through the removal of ubiquitin chains, enabling more robust induction of Tat expression for escape from latency to potentiate the effectiveness of antiviral regimens [66]. In the case of mitosis, BRISC has been found to promote the assembly of the functional bipolar spindle by controlling K63-linked ubiquitination of the nuclear mitotic apparatus (NuMA) [67]. The latest research suggests that BRISC can also regulate Janus kinase 2 (JAK2) activation and growth responses via the removal of JAK2 K63-linked ubiquitination, thereby attenuating JAK2 signaling-mediated expansion of hematopoietic stem cells [68].

### 4.5. Structural Basis of BRISC

According to the structures of the BRISC-SHMT2 complex (PDB ID: 6R8F, 6H3C) from *H. sapiens*, the mechanism of BRISC-regulated deubiquitination is investigated [87,88]. The higher-order assembly of BRCC36-Abro1 is considered essential for DUB activity and biological function [49]. Moreover, BRCC45 usually contains three domains: an N-terminal UEV-N domain, a C-terminal UEV-C domain, and a central RWD domain. Working as a bridge, the UEV-N domain of BRCC45 binds to Abro1 while the UEV-C domain binds to MERIT40 to assemble the BRISC complex [88]. SHMT2 is a vital metabolic enzyme in one-carbon metabolism catalyzing the conversion of serine to glycine, which has been reported to be essential for cell growth and proliferation [94]. To date, SHMT2 is the first reported endogenous BRISC inhibitor, which mainly prevents non-specific DUB activity of BRISC in cells [88]. The model of BRISC-SHMT2 cytokine signaling regulation is proposed (Figure 5C): generally, the formation of the BRISC-SHMT2 complex is necessary to enhance its delivery to ubiquitinated receptors such as IFNAR1/2 and HIV-1 Tat protein [65]. When the polyubiquitylated substrates are in close proximity, K63-linked ubiquitin chains may displace bound SHMT2 from BRISC. Then, BRCC36 deubiquitylates the K63-linked ubiquitin chains and limits the endocytosis and lysosomal degradation of the receptors [88]. Interestingly, pyridoxal-5′-phosphate (PLP), the active form of vitamin B6, is a cofactor of SHMT2 and can promote a shift in the SHMT2 oligomeric state from an inactive dimer to the enzymatically active tetramer [95]. It seems that only the inactive SHMT2 dimer, but not the active PLP-bound tetramer, is able to bind and inhibit BRISC [86,87,88]. Accordingly, the binding of PLP and SHMT2 regulates the BRISC–SHMT2 interaction and immune signaling in cells.

### 4.6. Functional Basis of BRCA1-A in DNA Damage Repair

In the nucleus, BRCC36, together with the subunits BRCC45/BRE, MERIT40, RAP80, and Abraxas, forms the BRCA1-A complex to participate in the regulation of DNA damage repair [87]. To maintain genomic integrity against various forms of DNA damage, especially the hazardous DNA double-strand breaks (DSBs), cells have evolved two major categories of DSB repair: homologous recombination (HR) and non-homologous end joining (NHEJ) [96,97]. The accuracy of HR, occurring only in the S/G2 phase, is conferred by using the sister chromatid as a template for a loss-free repair [98]. In contrast, throughout the cell cycle, NHEJ mediates direct ligation of the broken DNA ends without a homologous template as a low-fidelity pathway [99]. The tumor suppressor BRCA1 (breast cancer susceptibility protein 1) is regarded as a key regulator in HR repair and interacts with multiple distinct complexes, including BRCA1-A, BRCA1-B, BRCA1-C, and the BRCA1/PALB2/BRCA2 complex (Figure 5B) [100,101,102]. Of these, the binding of the BRCA1-A complex to BRCA1 is crucial for successful HR repair, which is recruited to the modified chromatin region in a manner dependent upon a cascade of phosphorylation and ubiquitination events (Table 1) [103,104].

In response to DSB, the MRN complex (Mre11-Rad50-NBS1) can rapidly recognize the DSB ends, triggering ataxia-telangiectasia mutated (ATM) kinase-dependent phosphorylation of histone H2AX (γH2AX) [105]. The γH2AX is, in turn, recognized by mediator of DNA damage checkpoint 1 (MDC1), subsequently recruiting the ubiquitin E3 ligases RNF8 and RNF168 together with the E2-conjugating enzyme UBC13 [106,107]. As a result, the histones H2A and H2AX are ubiquitinated with K63-linked polyubiquitin, providing a platform for the recruitment of more HR repair-related proteins [108,109]. Immediately after, the SUMO ligases PIAS1 and PIAS4 synthesize SUMO chains on various enzymes at DNA repair foci and these chains activate the SUMO-targeted ubiquitin ligase RNF4, which extends them as mixed SUMO-K63-linked ubiquitin chains [109,110]. As one of the BRCA1-A subunits, an important role of RAP80 is to recruit the BRCA1 to DNA damage sites to initiate DNA repair, which is ascribed to the effective recognition of RAP80 toward the SUMO-K63-linked ubiquitin chains [111]. Additionally, the accumulation of BRCA1-A at DNA repair foci contributes to the degradation of K63-linked ubiquitin chains in H2A/H2AX by another subunit BRCC36 in a DUB-dependent manner, which simultaneously reinforces the recruitment of BRCA1-A [69,112]. More importantly, when the repair is completed, BRCC36 has a role in rapidly removing DNA damage signals from chromatin to suppress hyperactive HR repair [87]. Finally, BRCA1-A can sequester BRCA1 away from other binding partners, such as BACH1 or CtIP, and withdraw it from the damage site to end the HR repair process [113,114]. There is evidence that the active site mutation of BRCC36 leads to increased ubiquitination at histone γH2AX, thus resulting in hyperactive HR-based gene conversion events and a hypersensitive response to genotoxic stress [69,115]. Therefore, the DUB activity of BRCC36 is considered important in promoting the stable accumulation of the BRCA1-A at DSB and modulating HR repair by preventing over-resection of DSB ends.

### 4.7. Structural Basis of BRCA1-A

Recently, Rabl et al. resolved the structure of the BRCA1-A complex (PDB ID: 6GVW) from *Mus musculus* to further study its structural basis in DNA damage repair [87]. The Abraxas subunit carries a nuclear import signal that is essential for nuclear localization of BRCA1-A while the binding of the Abraxas with BRCC36 confers DUB activity [49,116]. Then, BRCC45 interacts with Abraxas and MERIT40 through its UEV-N and UEV-C domains, respectively [87]. As an extended and largely unstructured subunit, RAP80 carries an N-terminus SIM-UIM domain, which is mainly responsible for recognizing and binding to the K63-linked polyubiquitin at DNA repair foci [86,117]. The C-terminus of BRCA1 contains a phosphopeptide-binding domain BRCT, which mainly interacts with phosphorylated proteins to form complexes [118]. When DNA damage occurs, the S404 and S406 sites of Abraxas are doubly phosphorylated (p-S404/p-S406) to provide a high-affinity docking cradle for BRCA1, thereby inducing the formation of BRCA1 dimerization (Figure 5D) [102,119]. Meanwhile, the interaction of BRCA1 with other subunits, such as BRCC45 or MERIT40, can place BRCA1 at the periphery of the BRCA1-A arc so as to not affect the binding of the BRCA1-A complex with ubiquitin chains [86,87,120].

### 4.8. Functional Basis of Rpn11 in Proteasome-Dependent Versatility

In eukaryotes, the 26S proteasome, consisting of the 20S core particle (CP) and one or two 19S regulatory particles (RPs), is a multi-subunit protease complex, which is primarily responsible for the degradation of many intracellular proteins with ubiquitin tags to maintain proteostasis in cells [121,122]. The 19S RP has three distinct DUB components: two cysteine proteases (USP14/Ubp6 and UCH37/UCH-L5) and a metalloprotease (Rpn11) [121,123]. Among them, by cutting at the base of the ubiquitin chain to release the chain en bloc, Rpn11 is believed to function in a dual capacity: (1) to facilitate substrate translocation into the 20S CP and result in proteolysis, and (2) release the substrate from the proteasome to escape degradation [29,124]. As a result, it has been suggested that Rpn11 works as a proteasomal proofreading device to determine the fate of incoming substrates [125].

So far, Rpn11 with DUB activity has been shown to function in diverse proteasome-dependent biological processes, including c-Jun stability, E2F transcription factor 1 (E2F1)-mediated tumor formation, human epidermal growth factor 2 (ErbB2) expression level, DNA repair, osteoclast and embryonic stem cell differentiation, and aggresome disassembly and clearance (Table 1) [70,71,72,73,74,126,127,128]. The AP1 transcription factor c-Jun, which plays significant roles in cell cycle and apoptotic pathways, is one of the many cellular proteins targeted for degradation by the proteasome. Therefore, tight control of the intracellular concentrations of active c-Jun is required, a process that is achieved through rapid turnover by E3 ligases Itch/SCF-mediated ubiquitination together with Rpn11-mediated deubiquitination [70]. Another similar example is the transcriptional factor E2F1, the hyperactivation of which frequently occurs in human cancers and contributes to malignant progression. Wang et al. demonstrated that Rpn11 efficiently deubiquitinated E2F1 by removing the K63-linked polyubiquitin chains, thus stabilizing the protein to promote tumorigenesis [71,129]. With respect to the oncogene receptor-tyrosine kinase ErbB2, Rpn11 can regulate its ubiquitylation to improve the stability in cancer cells [72]. Moreover, Rpn11 promotes the correct coordination of the cellular response to DSB: in NHEJ, it acts to restrain 53BP1 accumulation by countering both RNF8/RNF168-mediated histone K63-linked deubiquitination and JMJD2A chromatin eviction, and facilitating HR repair through the promotion of RAD51 loading [73]. Moreover, microphthalmia-associated transcription factor (Mitf) is deubiquitinated by Rpn11 in osteoclasts to allow more stable expression, which is essential for osteoclast differentiation [74]. Rpn11 is also crucial in maintaining the self-renewal and pluripotency of embryonic stem cells by controlling deubiquitination [126]. Interestingly, although the protein aggregates in the cell cannot be processed by the proteasome, Hao et al. presented evidence that free ubiquitin chains, produced by Rpn11, bound and activated the deacetylase HDAC6, subsequently stimulating autophagy-dependent aggresome disassembly and clearance [127].

### 4.9. Structural Basis of Rpn11

**Figure 6:**
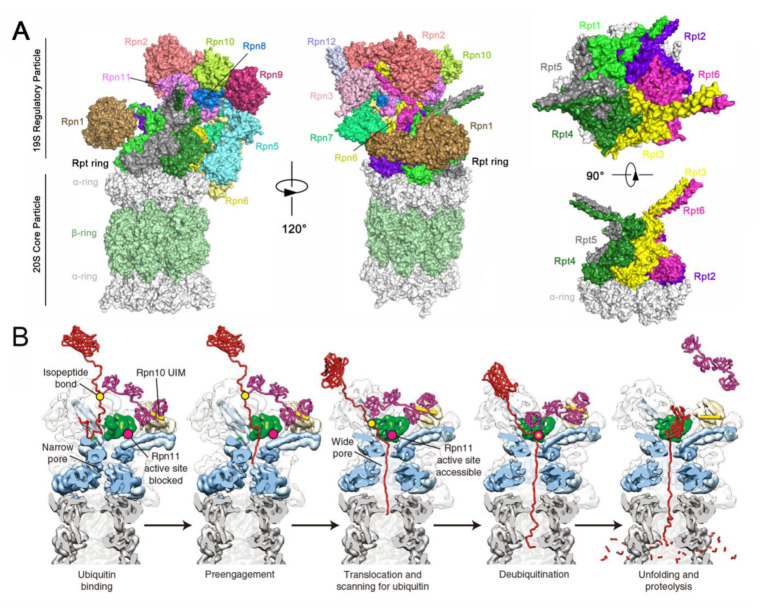
(**A**) High-resolution structure of human 26S proteasome. Figures were generated from PDB 5VFS. The α rings are in gray and the β ring is in pale green. The Rpn1-Rpn3 and Rpn5-Rpn12 are colored brown, orange, pink, cyan, yellow, green, blue, orange red, limon, magenta, and light blue, respectively. The Rpt1-Rpt6 are colored light green, purple, light yellow, dark green, dark gray, and violet, respectively. (**B**) Structure-based model for substrate engagement and degradation by the 26S proteasome. The substrate, ubiquitin chain, Rpn10 UIM, Rpn11, and N-ring with subjacent peptidase are colored red, purple, yellow cylinder, green, and gray, respectively. Panels **B** are adapted with permission from [136]. Copyright 2013 Springer Nature.

On the basis of the structure of the 26S proteasome (PDB ID: 5VFS) from *H. sapiens*, the 19S RP was found to be composed of 6 analogous ATPases (Rpt1-Rpt6), at least 13 extra integral subunits (Rpn1-Rpn13), and several transiently associated subunits, together stabilizing the 26S holoenzyme and providing substrate specificity (Figure 6A) [130,131]. Of these, three integral subunits, Rpn1, Rpn10, and Rpn13, as ubiquitin receptors, are primarily responsible for the initial recognition and binding of substrate [132]. Once bound, three corresponding DUBs, USP14, Rpn11, and UCHL5, rapidly disassemble most polyubiquitin linkages [36,133]. Intriguingly, both the USP14 and UCHL5 are only very transiently bound to the 19S RP during the degradation process while Rpn11 is an integral DUB in the proteasome, positioned directly above the substrate entry port of the 19S RP [134,135]. Another key feature of Rpn11 is the ATP dependence of its DUB activity [39,130]. After recruitment to the proteasome, the targeted substrates with ubiquitin tags are unfolded and translocated from the RP into the CP, which is followed by the removal of ubiquitin chains, a process that is driven by the heterohexamer ATPase motor.

Similar to other AAA+ ATPases, the Rpt subunits use ATP hydrolysis to undergo conformational changes and to exert mechanical force on the substrate [137]. Given this, the proteasome adopts four distinct conformational states (s1–s4) during processive substrate translocation [39,136]. The substrate-free proteasome predominantly adopts the s1 state, in which the Ins-1 loop is unstructured, with a low electron density and an extremely weak stabilization of the conformation. In contrast, the s3 state is the most heavily populated of the substrate-engaged states, where the Ins-1 loop switches from the inactive closed state to the active hairpin state and is strongly stabilized through interactions with Rpn5 [39]. Matyskiela et al. proposed a structure-based model for the substrate-engaged degradation by the 26S proteasome (Figure 6B). In this model, the ubiquitinated substrate is first tethered through its ubiquitin chain to the UIM of Rpn10. Then, the flexible substrate tail can enter the accessible N-ring pore and contact the uppermost subunits of the AAA+ domain spiral staircase. Upon substrate engagement, the Rpts become rearranged into a new spiral staircase and Rpn11 shifts to a central location directly above the N-ring pore, thus exposing its active site. Finally, all ubiquitin modifications are removed due to the DUB activity of Rpn11, thus facilitating fast translocation, unfolding, and degradation of the substrate [136].

### 4.10. Functional Basis of CSN5 in Regulating the Cullin-RING E3 Ubiquitin Ligases

The Cop9 signalosome (CSN), composed of six subunits with a PCI domain (CSN1–CSN4, CSN7, and CSN8) and two subunits with an MPN domain (CSN5 and CSN6), is a conserved eight-subunit complex, which functions in the ubiquitin-proteasome system by regulating the activity of cullin-RING E3 ubiquitin ligases (CRLs) (Table 1) [75,138,139]. As is well known, CRLs are the largest subfamily of all human E3 proteins (accounting for ~30%) and are responsible for 20% of proteasome-mediated protein degradation [140,141]. The activity of all CRLs is regulated by the ubiquitin-like activator NEDD8-mediated neddylation and deneddylation cycle, a process that is precisely dependent on the DUB activity of the CSN5 subunit in CSN [142,143]. Actually, elevated expression of CSN5 and other CSN subunits has been found in various human cancers, which is most probably indirect via the dysregulation of CRLs and the disturbed balance of oncogenes and tumor suppressors controlled by CRLs [144,145].

As the sole enzyme capable of removing NEDD8 modifications from CRLs, the CSN-mediated process positively and negatively affects many pathways, including cell cycle control, apoptosis, vascular morphogenesis, meiosis-to-mitosis transition, DNA repair, and oxygen homeostasis. For example, constitutive knockouts of various subunits in the CSN result in early embryonic death, featuring an accumulation of potential substrates of CSN-regulated CLRs, such as p27^kip1^, cyclin E, and p53, which contributes to impaired proliferation and accelerated apoptosis [146,147,148]. Meanwhile, several other proliferation-related proteins (e.g., p21^cip1^) and transcriptional regulators (e.g., SMAD4 and SMAD7), and some cyclins (cyclin D1, E, and B1) can be regulated by CSN-controlled CLRs [149,150,151,152]. Additionally, the CSN not only enhances cancer cell invasion and migration by blocking the ubiquitination and degradation of survivin and snail but also contributes to the escape of cancer cells from immune surveillance by stabilizing PD-L1 [153,154,155]. In addition, the CSN functions in conjunction with the myelocytomatosis oncogene (MYC) to modulate the transcription of many MYC target genes, such as Ccnd2 and E2f1, which are reactivated in breast cancer metastasis to promote cell proliferation, invasion, and angiogenesis [156,157]. Importantly, this process requires CSN-dependent deneddylation to modulate the activity of the CRL member SCF^skp2^, ultimately affecting the ubiquitylation of MYC. In some other studies, the CSN is thought to function in the regulation of protein stability through controlling the corresponding CRLs in various ways, including the HIF family of transcription factors HIF1-α by CRL2^VHL^ in mammalian oxygen homeostasis, and the substrate adaptor proteins Pop1p by SCF^Pop1p^ and Btb3p by Cul3-Btb3p [158,159]. Recently, the role of the CSN in DNA repair has been confirmed by regulating the activity of the CUL4-DDB1 family of CRLs, which are key players in DNA repair and maintenance of genome stability [157,160,161]. Usually, in the S phase or in response to DNA damage, the degradation of Spd1p enables the formation of active RNR enzymes in the cytosol, which stimulates the production of dNTPs [162]. This degradation process requires the CUL4-DDB1 ubiquitin ligase controlled by the CSN, together defining a DNA-damage-response pathway [161,163]. Another target protein that linked the CSN to DNA repair is CDT1, an important DNA replication licensing factor and a ubiquitylation substrate of SCF^skp2^ and/or CUL4-DDB1^CDT2^ [164]. During the S/G2 phase and after DNA damage, CDT1 is essential for the CSN to modulate CUL4-DDB1^CDT2^-mediated CDT1 ubiquitylation and degradation. In the case of UV-damaged DNA, CRL4^DDB2^, a member of the CRLs, containing CUL4, RBX1, DDB1, and DDB2, physically associates with the damaged DNA-binding protein (DDB), resulting in the overall decrease in UV-damaged DNA-binding activity [165]. This has been verified in experiments involving overexpressed CRL4^DDB2^ in human breast cancer and many other tumors, in which the abnormal expression causes a reduction in p48 levels, thus impairing the ability of DDB in lesion recognition and DNA repair in tumor cells [166,167]. Therefore, the CSN is considered to play a crucial role in the regulation of CRL4^DDB2^ activity through the deneddylation process [168]. Taken together, all these findings strongly qualify CSN5 as a potential drug target for anti-cancer therapy.

### 4.11. Structural Basis of CSN5

**Figure 7:**
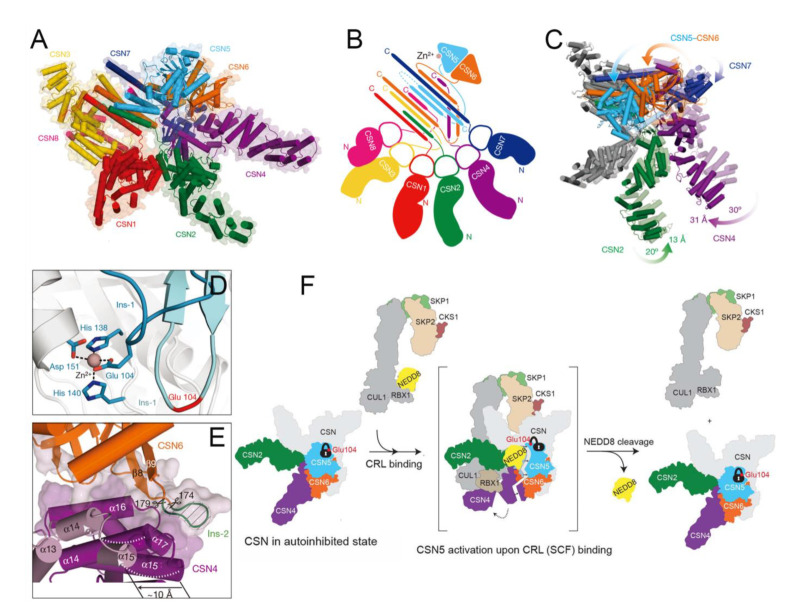
(**A**,**B**) Cryo-EM structure and flattened schematic of the CSN complex (PDB ID: 4D10). The CSN1-CSN8 are colored red, green, yellow, purple, blue, orange, deep blue, and pink, respectively. (**C**) Superimposition of CSN (PDB ID: 4D18 chains I-P, shade) and the CSN-CRL4A~N8^DDB2^ (EMD-3313) cryo-EM model. (**D**) Superposition of the Ins-1 loop with (cyan) or without (blue) an isopeptide-linked neddylated CRLs. In the absence of substrate, the CSN isopeptidase is inhibited by the CSN5 Ins-1 loop bearing Glu 104 and separately through the CSN4–CSN6 interface. (**E**) Removal of the CSN6 Ins-2 loop (green) disrupts the binding interface between CSN4 (purple) and CSN6 (orange) to impact the CSN5-CSN6 dimer. (**F**) Model of CRL-binding-induced conformational remodeling in CSN. The CSN2, CSN4, CSN5, CSN6, RBX1, SKP2, SKP1, CKS1, and NEDD8 are colored green, purple, cyan, orange, gray, sand, light green, and brown. Panels **A**, **B**, **D**, **E**, **F**, and **C** are adapted with permission from [34,168], respectively. Copyright 2014 Springer Nature, and copyright 2016 Springer Nature.

To date, several *H. sapiens* crystal structures of both the CSN (PDB ID: 4D10) and CSN-CRL complexes have been successfully resolved, including CSN-CRL2~N8 (PDB ID: 6R7F), CSN-SCF~N8^Skp2/Cks1^ (EMD-2173), CSN-SCF~N8^Fbw7^ (EMD-2174), and CSN-CRL4A~N8^DDB2^ (EMD-3313) [34,168,169,170]. From these structures, it can be observed that the N-terminal helical repeat domains of six PCI proteins (CSN1–CSN4, CSN7, and CSN8) radiate from the PCI ring to form the fingers of the splayed hand at the base of the complex (Figure 7A,B). Among them, the four longest subunits, CSN1–CSN4, are docked into the central region of the arc while the two shorter subunits, CSN7 and CSN8, serve as capping at each end [34]. Unlike the N-terminal, the extended C-terminal helix of each CSN1–CSN8 subunit forms a helical bundle while the CSN5 and CSN6 heterodimers with MPN domains adopt globular conformations just above the helical bundle [34,169].

Cavadini et al. presented the cryo-EM structure for CSN in a complex with neddylated CRL4A ligase (CSN-CRL4A~N8^DDB2^) to further elucidate the catalytic mechanism [168]. In the absence of a bound neddylated CRL substrate, the CSN isopeptidase is inhibited by the CSN5 Ins-1 loop bearing Glu 104 and separately through the CSN4–CSN6 interface (Figure 7D) [34]. Upon CRL4A~N8^DDB2^ substrate binding, substrate-induced conformational changes are observed in the CSN. The N-terminal arms of CSN2 and CSN4 move toward each other, and the expansion of the PCI ring pushes the N-terminal portion of CSN7 ~10 Å into the CSN6 MPN domain (Figure 7C). As a result, the CSN5-CSN6 dimer undergoes conformational rearrangement to facilitate movement toward the directed CRL4A^DDB2^ [168]. Furthermore, the removal of CSN6 Ins-2 has been shown to disrupt the binding interface between CSN4 and CSN6, contributing to enhanced deneddylase activity (Figure 7E) [34,170]. Combined with the structure of CSN-SCF~N8^Skp2/Cks1^, Lingaraju et al. proposed a model of CRL-binding-induced conformational remodeling in CSN (Figure 7F). First, CSN5 is auto-inhibited in the context of the holoenzyme by Glu 104 in the Ins-1 loop, coordinating to the catalytic Zn^2+^ ion. Then, with the binding of neddylated SCF^Skp2/Cks1^, topological conformation change occurs in the CSN5-CSN6 dimer, resulting in CSN5 activation. Finally, the activated CSN5 binds to NEDD8, leading to deneddylation from SCF^SKP2/CKS1^ via its deneddylase activity [34].

## 5. Currently Reported Inhibitors Targeting JAMMs

**Figure 8:**
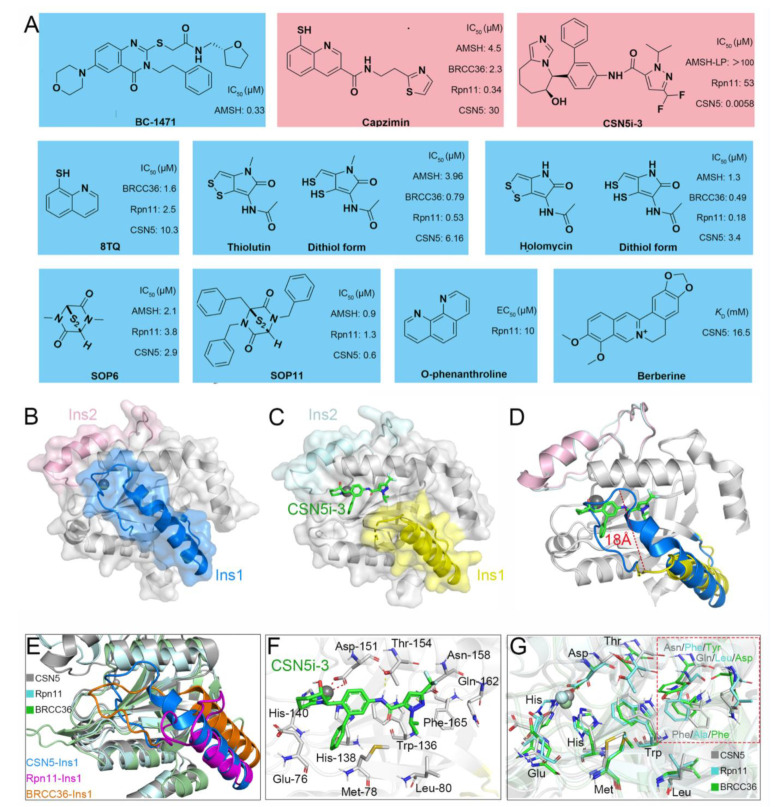
(**A**) Previously reported JAMM inhibitors. The Rpn11-specific inhibitor capzimin and CSN5-specific inhibitor CSN5i-3 are colored red. (**B**) Crystal structure of CSN5 (PDB ID: 4D10). The Ins-1 and Ins-2 are colored blue and pink, respectively. (**C**,**F**) Crystal structure of CSN5 in complex with CSN5i-3 (PDB ID: 5JOG). The Ins-1 and Ins-2 are colored yellow and palecyan, respectively. The CSN5i-3 is colored light green. (**D**) Superposition of the crystal structures of CSN5 and the complex of CSN5 and CSN5i-3. (**E**,**G**) Superposition of the crystal structures of CSN5, Rpn11, and BRCC36 (PDB ID: 6H3C). The CSN5, Rpn11, and BRCC36 are colored gray, cyan, and green, respectively. The Ins-1 regions of CSN5, Rpn11, and BRCC36 are colored blue, purple, and orange, respectively.

Recently, increasingly more attention has been placed on JAMMs and many inhibitors have been designed to target AMSH (BC-1471) [62], Rpn11 (8TQ, capzimin, thiolutin, holomycin, SOP6, SOP11, O-phenanthroline) [171,172,173,174], and CSN5 (Berberine, CSN5i-3) (Figure 8A) [42,175].

**AMSH.** Typically, after ubiquitination of the NALP7 inflammasome at Lys 288 and/or Lys 290, it is rapidly recruited by STAM for the subsequent endosomal passage and lysosomal degradation [176,177]. However, under the deubiquitination of AMSH, NALP7 is rescued from endolysosome sorting to permit inflammasome-dependent IL-1β cleavage [16]. Given this, by targeting the AMSH-ubiquitin-binding pocket, Bednash et al. performed a computer-assisted virtual screening using a library containing more than 500,000 experimental compounds [62]. As a result, a small molecule BC-1471 (IC_50_ = 0.33 μM) was observed to selectively decrease NALP7 abundance to suppress IL-1β release in several complementary human inflammatory systems, including THP-1 monocyte/macrophages, peripheral blood mononuclear cells, and lung organ culture. By evaluating BC-1471 against 38 other individual DUBs, mainly from cysteine families, the compound showed excellent specificity for AMSH because of no off-target DUB inhibition at the tested concentration. Afterwards, based on the molecular docking model of AMSH and BC-1471, the detailed mechanism of this interaction was further explored: Thr 63 formed electrostatic interactions with BC-1471, Tyr 105 formed π–π interactions with the benzene ring, and Val 97 formed σ–π interactions with the quinazoline ring. Together, these results indicate that BC-1471 exerts DUB inhibitory activity on AMSH to inhibit NALP7 inflammasome activity.

**Rpn11.** Multiple myeloma (MM) is a heterogeneous plasma cell malignancy for which there is currently no cure while the inhibition of the proteasome emerges as a powerful strategy for MM therapy [178,179]. Currently, the FDA has approved three medications, including bortezomib, carfilzomib, and ixazomib, to inhibit the proteasome by binding preferentially to the catalytic threonine residue of the β5 subunit within the 20S CP [180,181,182,183]. However, a proportion of patients do not respond to these compounds and those who do tend to relapse [184]. Therefore, there is an urgent need to develop new drugs targeting proteostasis with different mechanisms. Unlike the canonical proteasome inhibition, Rpn11-mediated inhibition occurs at 19S RP and thus may provide alternative opportunities to treat MM [29]. Importantly, it has been shown that Rpn11 is more highly expressed in patient MM cells while its loss-of-function by siRNA knockdown decreases MM cell viability [173].

In 2017, Li et al. and Perez et al. first identified a potent and selective moiety 8-thioquinoline (8TQ) (IC_50_ = 2.5 μM) by screening a library of metal-binding pharmacophores, which displayed strong inhibition of Rpn11 [174,185]. In addition, they demonstrated that 8TQ exerted its inhibitory activity through chelating the metal coordination of the active site Zn^2+^ ion. Unfortunately, 8TQ did not distinguish Rpn11 from other JAMMs, such as BRCC36 (IC_50_ = 1.6 μM) and CSN5 (IC_50_ = 10.3 μM). Subsequently, they performed structural optimization of 8TQ to further improve its inhibitory activity and selectivity. As a result, a lead compound capzimin was successfully developed, which showed potent activity for Rpn11 (IC50 = 0.34 µM) and high selectivity over other JAMMs such as AMSH (IC_50_ = 4.5 μM), BRCC36 (IC_50_ = 2.3 μM), and CSN5 (IC_50_ = 30 μM). Encouragingly, capzimin was equipotent against a set of bortezomib-sensitive and -resistant retinal pigment epithelial cells. Furthermore, capzimin also inhibited proliferation and induced apoptosis in several kinds of cancer cells, such as leukemia cells (SR and K562) and solid tumors (NCI-4460 and MCF7).

Initially, thiolutin (THL) was described as a dithiolopyrrolone antibiotic secreted by *Streptomyces*, and subsequently Lauinger et al. characterized it as a Zn^2+^ ion chelator capable of inhibiting DUB activity of several JAMMs, including AMSH (IC_50_ = 3.96 μM), BRCC36 (IC_50_ = 0.79 μM), Rpn11 (IC_50_ = 0.53 μM), and CSN5 (IC_50_ = 6.16 μM) [186]. Interestingly, THL exerted its activity only when it was reduced to the dithiol form inside the cell, which was similar to other dithiolopyrrolone antibiotics [187]. Moreover, the natural methyl derivative of THL, termed holomycin (HOL), was observed to inhibit Rpn11 (IC_50_ = 0.18 μM) and BRCC36 (IC_50_ = 0.49 μM) even more efficiently. Therefore, to a certain extent, THL and HOL might be regarded as selective inhibitors of BRCC36 and Rpn11. Afterwards, THL was used in the inhibition of BRCC36-mediated NLRP3 deubiquitination and activation by the Yin group [171]. They demonstrated that THL alleviated NLRP3-related inflammatory diseases in multiple mouse models, containing lipopolysaccharide-induced sepsis, monosodium urate-induced peritonitis, experimental autoimmune encephalomyelitis, CAPS, and methionine-choline-deficient diet-induced nonalcoholic fatty liver disease. As anticipated, HOL also displayed an even higher inhibitory activity against NLRP3 than THL. Molecular docking suggested that THL and HOL associated directly with Zn^2+^ ion of the BRCC36 active site, thus displacing the zinc-bound water molecule that was critical for the catalytic process. Additionally, the lower docking score of HOL (−6.392 kcal/mol) than THL (−5.169 kcal/mol) explained the reason for the higher inhibitory activity of HOL for BRCC36.

In 2018, Li et al. reported another type of Rpn11 inhibitor epidithiodiketopiperazines (ETPs), which were usually served as virulence factors generated from *Aspergillus fumigatus* secondary metabolites [172]. Among all tested ETPs, SOP6 was considered the core scaffold compound but did not show obvious selectivity between different JAMMs, such as AMSH (IC_50_ = 2.1 μM), Rpn11 (IC_50_ = 3.8 μM), and CSN5 (IC_50_ = 2.9 μM). Another ETP compound, SOP11, presented slightly higher inhibitory activity than SOP6 in AMSH (IC_50_ = 0.9 μM), Rpn11 (IC_50_ = 1.3 μM), and CSN5 (IC_50_ = 0.6 μM). Similar to capzimin, SOP11 not only triggered an unfolded protein response and induced an accumulation of polyubiquitin conjugates but also did not inhibit zinc-dependent enzymes unrelated to Rpn11, such as human carbonic anhydrase and matrix metalloproteinase 2 [172,174,185]. Meanwhile, the inhibition of proteasome by SOP11 had no effects on CSN5 activity, thus conferring it as a promising starting point to develop Rpn11 inhibitors. In another case of a known zinc chelator, O-phenanthroline (OPA) (EC_50_ = 10 μM) was also shown to induce apoptosis in MM cells and overcome resistance to the inhibitor bortezomib, which was ascribed to selectively inhibit Rpn11 activity without affecting other DUBs (e.g., USP1/USP2/USP4/USP5/USP7/USP8/USP20/UCH37) [173].

**CSN5.** CSN5 has been found to be overexpressed in a variety of cancers, including breast, thyroid, skin, ovarian, lung, and liver cancers [188], while the knockdown of CSN5 by siRNA has been shown to inhibit cell cycle progression and cause strong induction of apoptosis in hepatocellular carcinoma cells [189]. Because of this, stabilization of the neddylated CRLs through inhibition of CSN5 represents a novel therapeutic approach for the treatment of CSN5-dependent cancers. Based on the time-resolved fluorescence resonance energy transfer assay, researchers at Novartis developed a high-throughput screening platform for targeted screening of CSN5 inhibitors [42,190]. The final compound, CSN5i-3, inhibited CSN-mediated CRL deneddylation with an extremely low IC_50_ value (0.0058 μM) and showed a good pharmacokinetic profile. More importantly, CSN5i-3 had excellent selectivity since other JAMMs such as AMSH-LP (IC_50_ > 100 μM) and RPN11 (IC_50_ = 53 μM) were not or only weakly inhibited. In a panel of 500 cancer cell lines, CSN5i-3 exhibited varying degrees of inhibitory activities, and suppressed the growth of a human xenograft in mice.

Berberine (BBR), an isoquinoline quaternary alkaloid extracted from *Coptis chinensis*, has been used as a therapeutic agent in multiple diseases and presents strong anti-proliferative effects on cancer cells, such as breast, liver, and colorectal cancer cells [191,192,193]. By surface plasmon resonance assay, Liu et al. revealed a previously unrecognized antitumor mechanism of BBR: it could interact with CSN5 directly (*K*_D_ = 16.25 mM) to inhibit its deneddylase activity, therefore triggering the proteasome-dependent degradation of PD-L1 and activating the tumor-infiltrating T cells [175]. The reduction in PD-L1 expression in the tumor microenvironment subsequently attenuated the activation of immunosuppressive myeloid-derived suppressor cells (MDSCs) and regulatory T cells (Tregs), resulting in the antitumor effect in Lewis tumor xenograft mice. Collectively, BBR is a small-molecule inhibitor of CSN5 that disrupted PD-L1-mediated immunosuppression.

## 6. Challenges and Future Prospects

As early as 1987, Hershko et al. reported the first case of DUB inhibitor ubiquitin-aldehyde (UbAl), which was confirmed to oppose protein deubiquitination in reticulocyte lysate [194]. In the following decades, increasing evidence suggested that DUBs play significant roles in many human diseases, and, therefore, ambitious drug discovery and development efforts have been launched, resulting in more than 50 reported inhibitors [17,195]. In 2015, as a competitive inhibitor of proteasomal DUBs (preferring USP14 over UCHL5), VLX1570 became the first case applied in phase I trials for treating multiple myeloma and solid tumors, although it has been discontinued because of dose-limiting toxicity [196].

In recent years, their roles in multiple human diseases have made JAMMs attractive targets for therapeutic drugs. Additionally, extensive progress has been made in the development of JAMM-focused drug discovery. Nevertheless, there are still many unresolved issues and challenges.

First, JAMMs undergo substantial conformational changes upon substrate binding, suggesting flexibility of their active sites. This makes it challenging to predict efficient and specific inhibitors and design accurate tests to measure their efficiency. In fact, based on the *H. sapiens* crystal structures of CSN5 (PDB ID: 4D10) and the complex of CSN5 and CSN5i-3 (PDB ID: 5JOG), it can be clearly observed that the Ins-1 loop is deflected about 18 Å upon CSN5i-3 binding while other parts exhibit an excellent overlap (Figure 8B–D) [34,42]. In addition, by combining docking and molecular dynamic simulation, Kumar et al. revealed that the Ins-1 regions of Rpn11 and CSN5 show different flexibility, which, in turn, affects the binding of substrate [197]. Moreover, most of the residues in the Ins-1 loop region are not conserved, resulting in distinct structural differences (Figure 8E). Thus, we are reasonably confident that the Ins-1 region together with its conformational change will be important and must be considered in the design of efficient and selective JAMM inhibitors.

Second, JAMMs exhibit similar catalytic pockets that increase the difficulty of inhibitors’ specific targeting. The current studies demonstrate that the strong coordination of inhibitors with the Zn^2+^ ion in a mono- or bidentate fashion plays a key role in exerting their inhibitory activity against JAMMs. Specifically, according to the crystal structure of the CSN5 and CSN5i-3 complex (PDB ID: 5JOG), the tetrahydroimidazoazepinol moiety of the CSN5i-3 forms a coordination bond with the Zn^2+^ ion of the active site [42]. Meanwhile, CSN5i-3 also forms hydrogen bonds with Thr 154 and Asn 158, respectively (Figure 8F). Because the crystal structure of the Rpn11-capzimin complex is still not available, Kumar et al. performed molecular docking analysis and suggested that capzimin not only interacts with the Zn^2+^ ion of Rpn11 in a bidentate manner but also forms a stable hydrogen bond with the residue Thr 129 [197]. Then, how can a specific inhibitor be designed to bind to one JAMM only? After superposition of the *H. sapiens* crystal structure of CSN5, Rpn11, and BRCC36, we find that the majority of amino acids near the active site are highly overlapping. However, three amino acids are obviously different, including Asn 158, Gln 162, and Phe 165 of CSN5; Phe 133, Leu 136, and Ala 140 of Rpn11; and Tyr 142, Asp 146, and Phe 149 of BRCC36 (Figure 8G). Therefore, in addition to the consideration of the interaction between the inhibitor and Zn^2+^ ion, these different amino acids are also crucial in determining the selectivity of JAMM inhibitors.

Finally, only the crystal structure of CSN5 and inhibitor complex has been reported currently, whereas the protein-inhibitor complexes of all other JAMMs have not been resolved. As we know, a reliable conformational analysis of receptor–ligand interaction is significantly important for successful drug design [17]. Moreover, further studies are needed to clarify the structure–function relationship of some other JAMM family members, such as MYSM1 and eIF3f, which are not mentioned in detail in this review. For example, the role of MYSM1 as a transcriptional regulator of hematopoiesis and immune cell development has been demonstrated while the crystal structure of full-length MYSM1 remains unavailable, thereby impeding further drug discovery processes [198]. Besides, eIF3f is the core subunit with DUB activity in the eukaryotic initiation factor eIF3. Studies have confirmed that a decrease in eIF3f expression contributes to cancer development, but the molecular mechanism has not yet been fully elucidated [28]. With the development of technique and continued research, and more JAMM crystal structures being obtained, we believe that further exciting developments in the arenas of JAMM biology and drug discovery will be obtained in the future.

## 7. Conclusions

Given the growing evidence showing the importance of DUBs in the occurrence and development of many diseases, they are becoming a promising target for disease therapy and have attracted much attention from the pharmaceutical industry. Here, focusing on the JAMM family of DUBs, we provide a detailed discussion of their structural basis, catalytic mechanism, diverse functions, and currently reported inhibitors targeting AMSH, AMSH-LP, BRCC36, Rpn11, and CSN5. This review provides a deeper understanding of the molecular mechanism of JAMMs to develop new specific medicines based on the pathogenesis of different diseases.

## Figures and Tables

**Table 1:** List of JAMMs mentioned in this review and their functional roles.

Proteins	Functional Complex	Target Protein	Linkage Type	Regulation Effects	Ref.
AMSH	N/A	EGFR	K63-	Promote the recycling of EGFR	[59]
AMSH	N/A	Cx43	K63-	Protect gap junctions from degradation to regulate the intercellular communication	[60]
AMSH	N/A	NALP7	K63-	Lead to the inflammasome-dependent IL-1β cleavage and release	[62]
AMSH	N/A	CXCR4	Mono-	Regulate the stability and trafficking of CXCR4	[61]
AMSH	N/A	PAR_2_	Mono-	Regulate the trafficking and down-regulation of PAR_2_	[57]
AMSH	N/A	DOR	Mono-	Regulate the downregulation of the DOR	[58]
BRCC36	BRISC	NLRP3	K63-	Activate NLRP3 and promote inflammasome assembly	[64]
BRCC36	BRISC	IFNAR1/2	K63-	Promote the cellular response to Type I interferons	[65]
BRCC36	BRISC	HIV-1 Tat	K63-	Rescue Tat from destruction to potentiate the effectiveness of antiviral regimens	[66]
BRCC36	BRISC	NuMA	K63-	Promote the assembly of functional bipolar spindle during mitosis	[67]
BRCC36	BRISC	JAK2	K63-	Limit hematopoietic stem cell expansion	[68]
BRCC36	BRCA1-A	H2A/H2AX	K63-	Suppress hyperactive HR repair	[69]
Rpn11	26S proteasome	c-Jun	K48-	Maintain a stable intracellular concentration of c-Jun	[70]
Rpn11	26S proteasome	E2F1	K63-	Stabilize E2F1 protein to promote tumorigenesis	[71]
Rpn11	26S proteasome	ErbB2	ND	Regulate ErbB2 ubiquitylation and stability in cancer cells	[72]
Rpn11	26S proteasome	H2A/H2AX	K63-	Promote the correct coordination of the cellular response to DSB	[73]
Rpn11	26S proteasome	Mitf	ND	Allow more stable Mitf expression in osteoclast differentiation process	[74]
CSN5	Cop9 signalosome	CRLs	NEDD8	Maintain the proper activity of CRLs in myriad cellular processes	[75]

N/A: Not applicable; ND: Not determined.

## Data Availability

Not applicable.

## Abbreviations

DUB	Deubiquitinating enzyme
JAMM	JAB1/MPN/Mov34 metalloenzymes
AMSH	Associated molecule with the SH3 domain of STAM
AMSH-LP	AMSH-like protein
BRCA1	Breast cancer susceptibility protein 1
BRCC36	BRCA1-BRCA2-containing complex subunit 36
Rpn11	Regulatory particle non-ATPase 11
CSN	COP9 signalosome
SUMO	Small ubiquitin-like modifier
NEDD8	Neuronal precursor cell-expressed developmentally downregulated protein 8
USP	Ubiquitin-specific peptidase
OTU	Ovarian tumor protease
MINDYs	Motif interacting with ubiquitin-containing novel DUB family
UCH	Ubiquitin C-terminal hydroxylase
MJD	Machado-Josephin domain protease
ZUP1	Zinc finger-containing ubiquitin peptidase 1
ZnF	Zinc finger domain
UIM	Ubiquitin-interacting motif
UBA	Ubiquitin-associated domain
eIF3h	Eukaryotic translation initiation factor 3 subunit H
MYSM1	Myb-like, SWIRM, and MPN domains 1 protein
MPN	Mpr1/Pad1 N-terminal
SH3	Src-homology domain 3
STAM	Signal transducing adapter molecule
ESCRT	Endosomal sorting complexes required for transport
EGFR	Epidermal growth factor receptor
GPCR	G protein-coupled receptor
NALP	NACHT, LRR and PYD domains-containing protein
SBM	SH3 binding motif
CHMP	Charged multivesicular body proteins
MIM	MIT-interacting motif
MIT	Microtubule interacting and transport
UIM	Ubiquitin-interacting motif
VHS	Vps27/Hrs/STAM
ASC	Apoptosis-associated speck-like protein containing a caspase recruitment domain
SHMT2	Serine hydroxymethyltransferase 2
JAK2	Janus kinase 2
PLP	Pyridoxal-5′-phosphate
DSB	DNA double-strand breaks
HR	Homologous recombination
NHEJ	Non-homologous end joining
CP	Core particle
RPs	Regulatory particles
Mitf	Microphthalmia-associated transcription factor
CRLs	Cullin-RING E3 ubiquitin ligases
PD-L1	Programmed death-ligand 1
MYC	Myelocytomatosis oncogene
